# Computation‐Guided Tunnel Engineering Enhances O_2_ Transport and H_2_O_2_ Resistance of Fe(II)/α‐Ketoglutarate‐Dependent Dioxygenases

**DOI:** 10.1002/advs.76328

**Published:** 2026-07-06

**Authors:** Huan Liu, Lunjie Wu, Liying Mao, Songyin Zhao, Hu Liu, Shiyi Xin, Donglin Zhao, Jie Gu, Yan Xu, Xinye Wang, Yao Nie

**Affiliations:** ^1^ Lab of Brewing Microbiology and Applied Enzymology School of Biotechnology The Key Laboratory of Industrial Biotechnology Key Laboratory of Industrial Synthetic Biology of Jiangsu Province Ministry of Education Jiangnan University Wuxi China; ^2^ Institute of Biopharmaceutical and Health Engineering Tsinghua Shenzhen International Graduate School Tsinghua University Shenzhen China; ^3^ School of Life Sciences Ludong University Yantai Shandong China; ^4^ State Key Laboratory of Food Science and Technology Jiangnan University Wuxi China

**Keywords:** Fe(II)/α‐ketoglutarate dependent dioxygenase, protein structuromics, random acceleration molecular dynamics, small‐molecular tunnel, tunnel engineering

## Abstract

Fe(II)/α‐ketoglutarate‐dependent dioxygenases (αKGDs) are versatile biocatalysts whose catalytic activity relies on an efficient supply of O_2_ and αKG. However, in engineering applications, the O_2_ supply is seldom considered, whereas αKG is typically supplied through the oxidation of l‐glutamate by l‐glutamate oxidase, which concomitantly produces H_2_O_2_ that potently inhibits αKGD activity. This study developed a tunnel engineering strategy based on random accelerated molecular dynamics and protein structuromics to precisely modulate the access of small molecules (O_2_ and H_2_O_2_) to isoleucine dioxygenase (IDO), enhancing O_2_ transport while resisting H_2_O_2_ transport. Notably, mutant 1 (L179C/V225F/I240V) and mutant 2 (N193S/V225I/I240V) displayed markedly higher catalytic activity for five aliphatic amino acid substrates (Leu, Nle, Nva, Met, and Ile), reaching 27.2‐fold greater activity. In addition, mutant 2 exhibited 7.1‐fold higher activity against Ile in the presence of H_2_O_2_. This study highlights O_2_ and H_2_O_2_ tunnel engineering as a new strategy for enhancing αKGDs activity and antioxidative properties.

## Graphical abstract

This study presents a computational tunnel engineering strategy in Fe(II)/α‐ketoglutarate‐dependent dioxygenases (αKGDs) to enhance oxygen (O_2_) transport, an essential molecule for αKGDs, and limit hydrogen peroxide (H_2_O_2_) entry, an inhibitor from the αKG supply system. Engineered small‐molecule tunnel mutants exhibit enhanced activity of aliphatic amino acids and improved H_2_O_2_ resistance, providing a novel strategy for optimizing enzyme performance and oxidative stability.

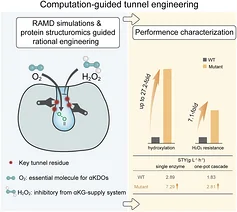

## 1 Introduction

Fe(II)/α‐ketoglutarate‐dependent dioxygenases (αKGDs) comprise a remarkably diverse enzyme family found ubiquitously in prokaryotic and eukaryotic organisms, responsible for catalyzing extensive oxidative transformations essential for numerous biological processes [1, 2, 3]. αKGDs exhibit excellent catalytic versatility by activating molecular oxygen to facilitate reactions such as hydroxylation, demethylation, cyclization, rearrangement, desaturation, and epoxidation [1, 2, 3]. The catalytic prowess of αKGDs stems from their ability to generate highly reactive iron–oxygen species (Fe(IV)‐oxo) capable of abstracting hydrogen atoms from inert C–H bonds, thereby enabling functionalization at positions typically recalcitrant to chemical modification [4]. This extraordinary biocatalytic capability has positioned αKGDs as invaluable tools in synthetic biology and biocatalysis for producing high‐value compounds, including pharmaceutically relevant natural products [5, 6, 7, 8]. modified amino acids [9, 10, 11, 12, 13], and fine chemicals [14, 15]. The continuous exploration of αKGDs has expanded the frontiers of enzymatic synthesis and catalysis, facilitating their utilization in industrial‐scale biomanufacturing processes.

The catalytic activities of αKGDs involve the coordinated actions of multiple ligand molecules. These include Fe(II), αKG, O_2_, and the target substrates. Fe(II) is positioned at the core of this catalytic system, providing the chemical basis for the remarkable catalytic diversity of this enzyme family. During the catalytic cycle, O_2_ binds the Fe(II) center, triggering the oxidative decarboxylation of αKG to succinate and CO_2_ while simultaneously generating a high‐spin Fe(IV)‐oxo intermediate with extraordinary reactivity [4]. Complementary to the crucial role of Fe(II), the co‐substrate αKG profoundly influences the catalytic outcome [16, 17, 18, 19]. However, limited attention has been paid to O_2_, another small molecule involved in the reaction. Recent data suggest that engineering a gas tunnel could enhance the O_2_‐sensing capacity of PHD2 [20], emphasizing the potential of O_2_ transport in regulating the catalytic efficiency of αKGDs.

Additionally, although αKGDs have gained momentum for industrial applications owing to their excellent catalytic capabilities, one of the challenges in scaling up these processes is securing the cost‐effective supply of αKG. Typically, l‐glutamate oxidase (LGOX) is employed to generate the essential co‐substrate αKG from relatively inexpensive glutamate, with catalase (katG) added to remove the LGOX‐derived byproduct, H_2_O_2_, a potent inhibitor of αKGD activity [21, 22, 23]. Recently, the self‐assembly of LGOX and katG was explored to enhance their spatial proximity and maximize the in situ elimination of H_2_O_2_ [24]. However, the inevitable leakage of H_2_O_2_ remains a long‐standing and critical issue for large‐scale applications of αKGDs.

The above content raises two critical scientific concerns for αKGD‐based biosynthesis: (1) how to efficiently transport O_2_ to enhance the performance of αKGDs and (2) how to avoid inhibiting αKGD activity by H_2_O_2_ in multi‐enzyme cascade systems. Both issues are fundamentally related to the transport pathways of small molecules within the protein matrix. However, these pathways remain largely uncharacterized and underexploited in enzyme engineering efforts. Notably, insights gained from computational tools such as molecular dynamics (MD) simulations have greatly accelerated protein engineering processes [25, 26, 27, 28, 29]. Specifically, transport tunnels for organic molecules (including substrates and products) can be extensively studied through random acceleration molecular dynamics (RAMD) simulations [30, 31, 32, 33, 34]. The primary focus of this investigation was to design tunnels, using enhanced sampling, random accelerated MD simulations, and protein structuromics to achieve a dual objective: enhancing O_2_ access to the active site while impairing H_2_O_2_ permeation into the active site pocket. Subsequent iterative mutagenesis and kinetic profiling identified three‐point mutants with increased catalytic activity and H_2_O_2_ tolerance, highlighting the importance of small‐molecule tunneling in αKGD catalysis.

## 2 Results and Discussion

### 2.1 Prediction of Potential Key Sites in O_2_ and H_2_O_2_ Tunnels

Characterization of transport tunnels for small molecules such as O_2_ and H_2_O_2_ within enzyme structures remains challenging because of their small sizes and transient behaviors. Herein, an enhanced sampling simulation was employed to comprehensively explore the transport pathways of O_2_ and H_2_O_2_ in isoleucine dioxygenase (IDO). Although the apo structure of IDO has been resolved (PDB ID: 6LNH), the absence of available enzyme–ligand complex structure remains a challenge for further investigation. Therefore, we used artificial intelligence (AI)‐based modeling tools to construct IDO–ligand complex structures, both of which exhibited a 6‐coordinate structure centered on the ferrous ions (Figure S1A). After energy minimization and MD equilibration, RAMD simulations were conducted using O_2_ and H_2_O_2_ as the target molecules. The simulated trajectories revealed multiple potential transport pathways for each molecule, with nine predominant tunnels identified in both cases (Figure S1B). To quantitatively evaluate tunnel usage, the tunnel frequency was defined as the percentage of independent RAMD trajectories in which the small molecule successfully exited the active site through a specific tunnel. Notably, the tunnels for both O_2_ and H_2_O_2_ were highly similar, likely due to their comparable dimensions. Nevertheless, distinct tunnel preferences were observed (Figure 1A): the top three tunnels for O_2_ were primarily located within the DSBH fold (T1/T2/T4), whereas those for H_2_O_2_ were mainly located at the active site pocket and loop regions (T4/T6/T8). These findings suggest that, although O_2_ and H_2_O_2_ might share similar transport tunnel networks, they might exhibit different tunnel‐usage preferences influenced by specific interactions between the small molecules and the residues lining the tunnels.

**FIGURE 1:**
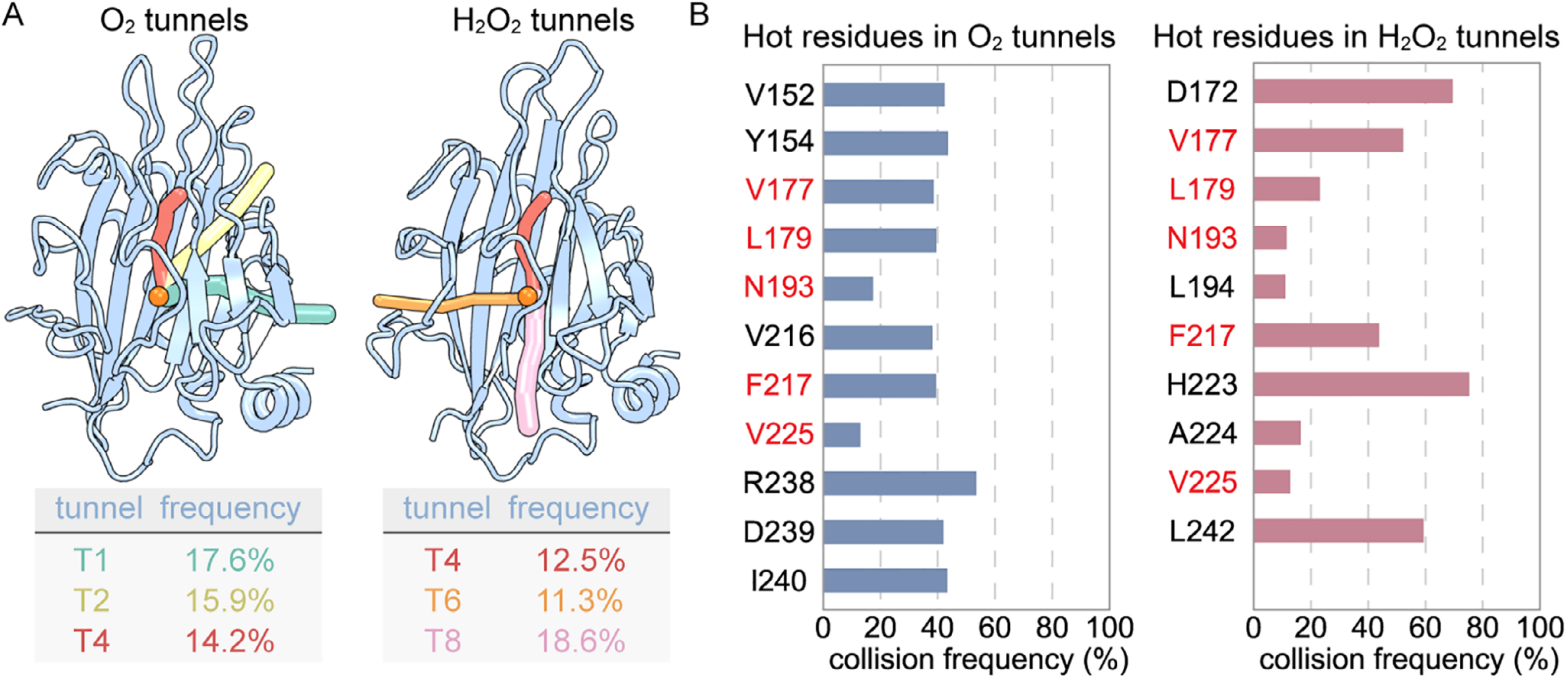
Analysis of O_2_ and H_2_O_2_ transport tunnels and hotspot residues in IDO. (A) The top three O_2_ (left) and H_2_O_2_ (right) transport tunnels identified via RAMD simulations. (B) Hotspot residues within the O_2_ and H_2_O_2_ transport tunnels show >10% collision frequency in the simulations. The residues highlighted in red represent hotspots shared by both O_2_ and H_2_O_2_ tunnels.

In addition, we identified key residues within the tunnels. During RAMD simulations, external forces in arbitrary directions were applied to the target molecules (O_2_ or H_2_O_2_) to facilitate tunnel exploration. Instances of proximity between the small molecules and tunnel‐lining residues (e.g., <3 Å), resembling collision‐like events, were observed during the simulations. Accordingly, we analyzed the “collision” frequency between the molecules and tunnel residues. Using a collision frequency threshold of 10%, 15 tunnel‐lining residues were identified as being potentially involved in small‐molecule transport (Figure 1B), comprising eight residues predominantly associated with O_2_, seven predominantly associated with H_2_O_2_, and five (V177, L179, N193, F217, and V225) involved in both O_2_ and H_2_O_2_ transport routes. Consequently, we designated these critical tunnel lining residues as candidate sites for subsequent tunnel engineering, excluding those involved in substrate/co‐substrate binding and the catalytic triad (Y154, V216, R238, D239, and H223).

### 2.2 Conservation Analysis of Tunnel Residues Based on Protein Structuromics

Conservation analysis of amino acid residues aids in refining the scope of site‐directed mutagenesis [33, 35]. Multiple‐sequence alignment, a conventional method for assessing the conservation of functionally important residues, has been highly effective in guiding protein engineering strategies. To examine the conservation of the key tunnel‐lining residues identified in IDO within the PF10014 family, we performed phylogenetic analysis and identified five distinct clusters (Figure 2A). However, the PF10014 family exhibited low sequence identity (Figure 2B), with approximately 80% pairwise alignments below 30%, which reduced our confidence in the residue‐conservation analysis. For protein families with high sequence diversity, such as the PF10014 family, protein structuromics is a promising alternative for analyzing the conservation of functional residues [36, 37]. Before large‐scale structural alignment, we performed sequence‐based and structure‐based alignments of representative proteins from each cluster, demonstrating higher accuracy in structure‐based results (Figures S2 and S3).

**FIGURE 2:**
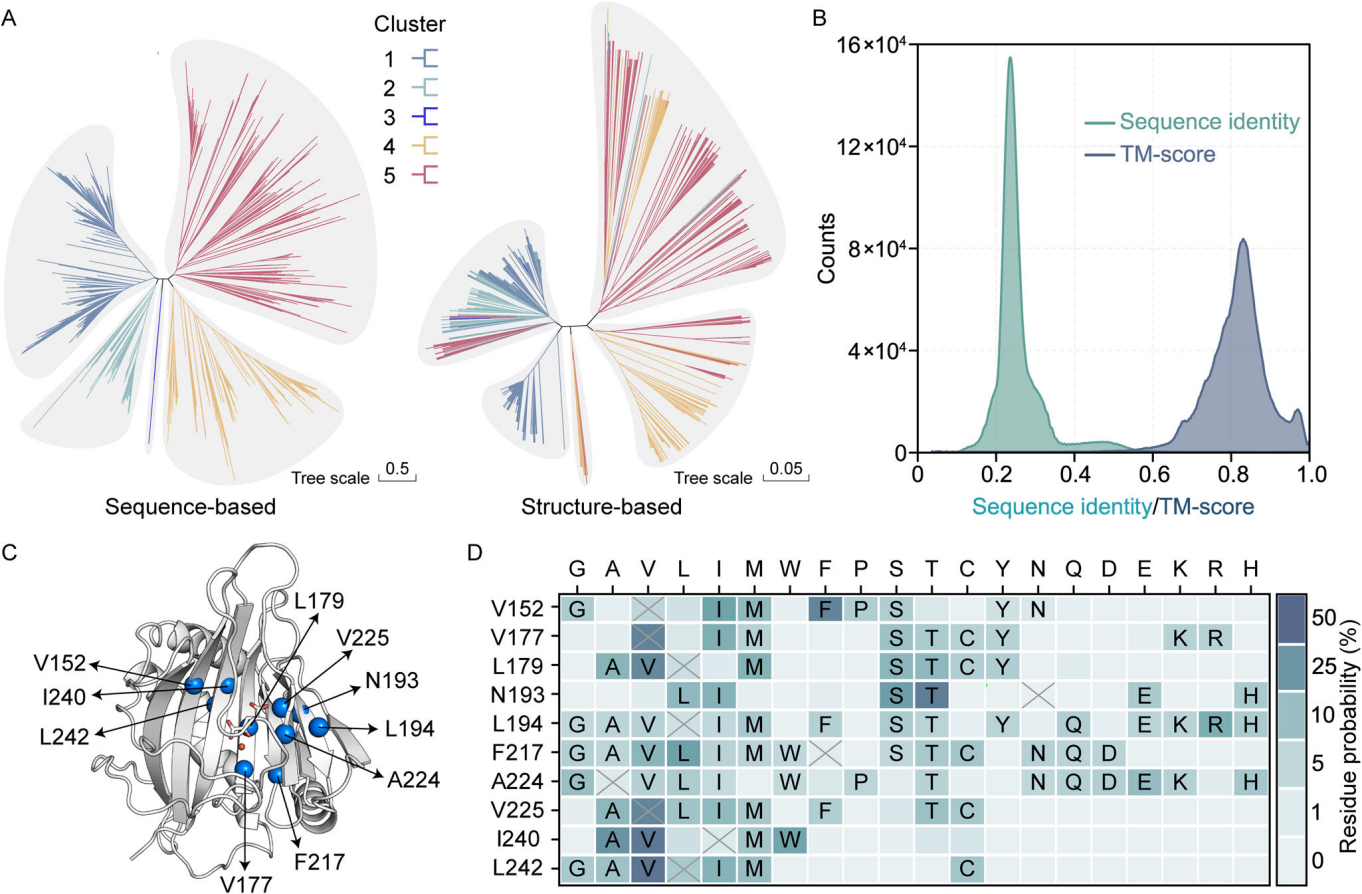
Conservation of hotspot residues in tunnels. (A) Phylogenetic analysis of the IDO family member, PF10014, based on its sequence (left) and structure (right). (B) Distribution of sequence and structural similarity. (C) Mutation sites in IDO. (D) Conservation of hotspot residues (>10% occurrence, marked in black) based on the structural alignment for the mutation library design.

Therefore, conservation analysis of key tunnel‐lining residues was conducted via structure‐based alignments using TM‐align. Structural alignment revealed remarkable structural similarity among these family members, as nearly all pairwise TM‐scores exceeded 0.5, with over 99% of scores ranging from 0.6 to 1.0 (Figure 2B). TM‐scores above 0.5 typically indicate that two proteins share the same fold [38]. Simultaneously, we constructed a structure‐based phylogenetic tree (Figure 2A), which exhibited substantially different cluster classifications than the sequence‐based approach did. Specifically, cluster 1, defined by sequence‐based classification, was distributed among different structural clusters, and similar patterns were observed with clusters 3 and 5. We used the structural‐alignment results to assess the conservation of key tunnel‐lining residues (Figure 2C,D). Highly conserved residues and residues directly involved in catalysis were excluded from mutagenesis. For the remaining candidate sites, substitutions for site‐directed mutagenesis were selected based on a 10% amino acid frequency threshold, in which only residues with frequencies ≥10% in the structural alignment were considered evolutionarily acceptable substitutions.

### 2.3 Library Screening Results and Hit Verification

Based on the O_2_ and H_2_O_2_ tunnel‐analysis results and structural alignment of key sites, a site‐directed mutagenesis library comprising 86 mutants was constructed targeting residues V152, V177, L179, N193, L194, F217, A244, V225, I240, and L242 according to the substitution‐selection criteria described in 2.2 section. We evaluated the catalytic activities of these mutants toward 5 substrates (Leu, Nle, Nva, Met, and Ile; denoted as **S1**–**S5**, respectively) to investigate the influence of the tunnel residues on the catalytic activity of IDO. In addition, using Ile as a representative substrate, we added 10 mm H_2_O_2_ to assess the residual activity, and analyzed the effect of tunnel engineering on the H_2_O_2_ resistance of IDO. Given that the co‐substrate αKG serves as a key intermediate in the TCA cycle, all activity analyses were conducted with purified enzymes to strictly quantify the αKG concentration in the system and ensure accurate activity measurements. We monitored the formation of hydroxylated products via high‐performance liquid chromatography (HPLC) analysis to characterize the mutant activity (Figure 3A).

**FIGURE 3:**
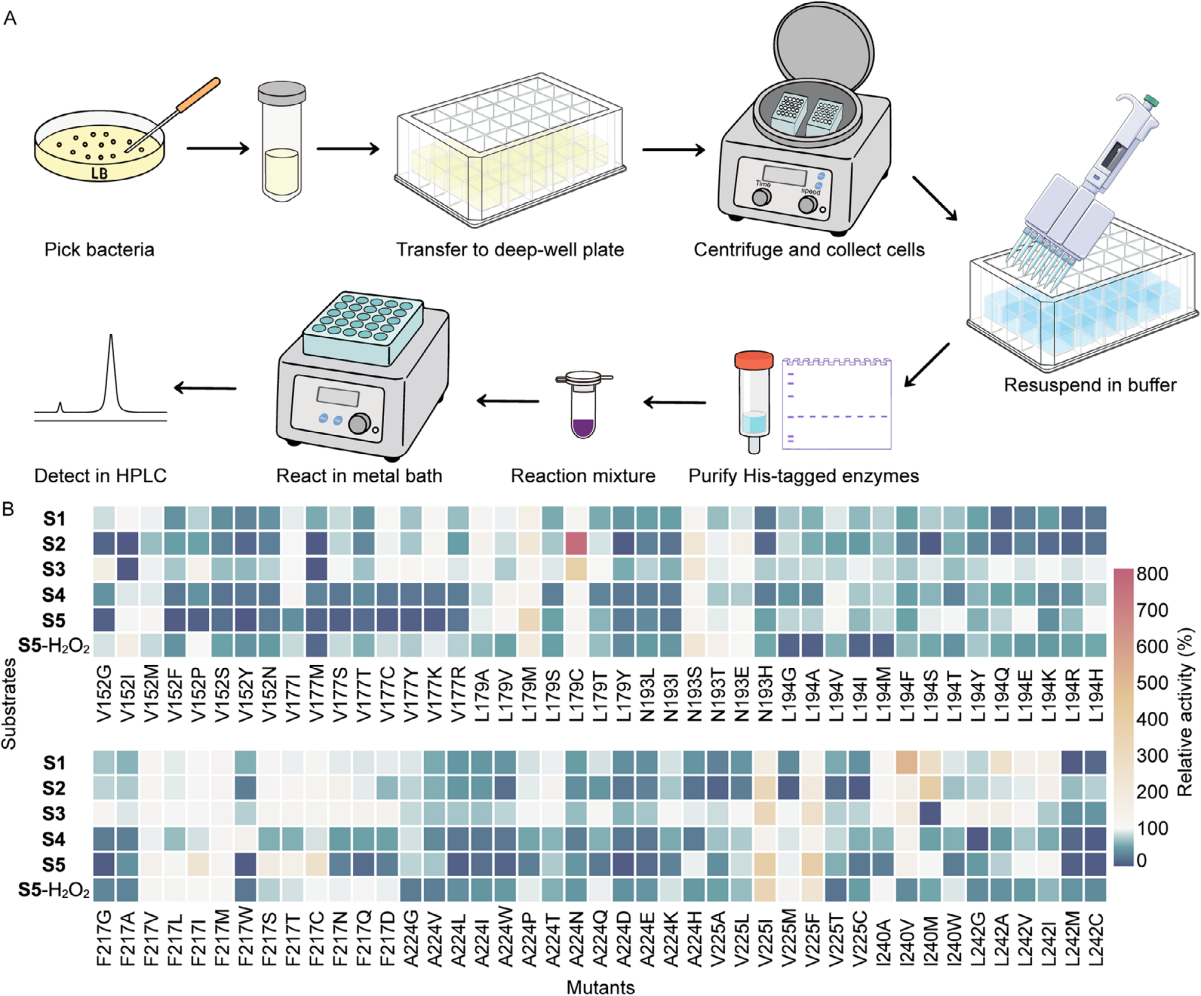
Screening of the tunnel hotspot mutant library. (A) Workflow of the process followed for tunnel hotspot mutant library screening. B) Relative catalytic activity of tunnel hotspot mutants toward **S1**–**S5** and **S5** with H_2_O_2_ normalized to WT activity (100%).

By screening the single‐point mutant library, we identified five mutants (L179C, N193S, V225I, V225F, and I240V) with significantly higher activity toward all five substrates, demonstrating broad‐spectrum catalytic improvement while maintaining H_2_O_2_ resistance. Notably, V225I displayed 3.4‐fold higher residual activity than the wild‐type (WT) under H_2_O_2_ stress conditions (Figure 3B).

Given the effectiveness of tunnel engineering in enhancing the hydroxylation activity and H_2_O_2_ resistance, we performed iterative mutagenesis. We selected single‐point mutants exhibiting the highest activity toward each of the five substrates and the greatest H_2_O_2_ resistance and combined them with other beneficial mutations to obtain optimal double mutants. In the third round, we used these double mutants as templates for iterative combinations with other beneficial mutations. We terminated the iterative process when no further improvement in mutant activity was observed. Through this mutagenesis strategy, combinatorial mutants were screened (Figures S4–S10), leading to the identification of M1 (L179C/V225F/I240V) and M2 (N193S/V225I/I240V) as the optimal mutants. M1 exhibited the highest catalytic activity toward substrates **S1**–**S4**, with 6.0‐, 19.7‐, 27.2‐, and 3.2‐fold improvements over the WT, respectively (Figure 4A–D), whereas M2 showed the highest activity toward Ile and the strongest H_2_O_2_ resistance, with 3.1‐ and 7.1‐fold improvements, respectively (Figure 4E). Therefore, M1 and M2 were selected to represent the evolutionary pathways leading to the optimal mutants for different substrates (Figure 4). Notably, in the S5‐H_2_O_2_ assay, both the substrate and H_2_O_2_ concentrations were 10 mm, and additional activity assays under increasing H_2_O_2_ concentrations (0–20 mm) further confirmed the enhanced oxidative resistance of M1 and M2, particularly M2 (Figure S11). Although the optimal mutant for each substrate evolved from different single‐point mutations, the highly repetitive mutation sites among the top‐performing mutants suggested that the increase in activity reflected a conserved mechanism (i.e., all activity increases are primarily attributable to improved O_2_ transport efficiency) rather than substrate‐specific effects. Overall, M1 and M2 exhibited significantly higher activity with **S1**–**S5**, accompanied by improved resistance to H_2_O_2_, confirming their functional enhancement relative to the WT (Figure 5).

**FIGURE 4:**
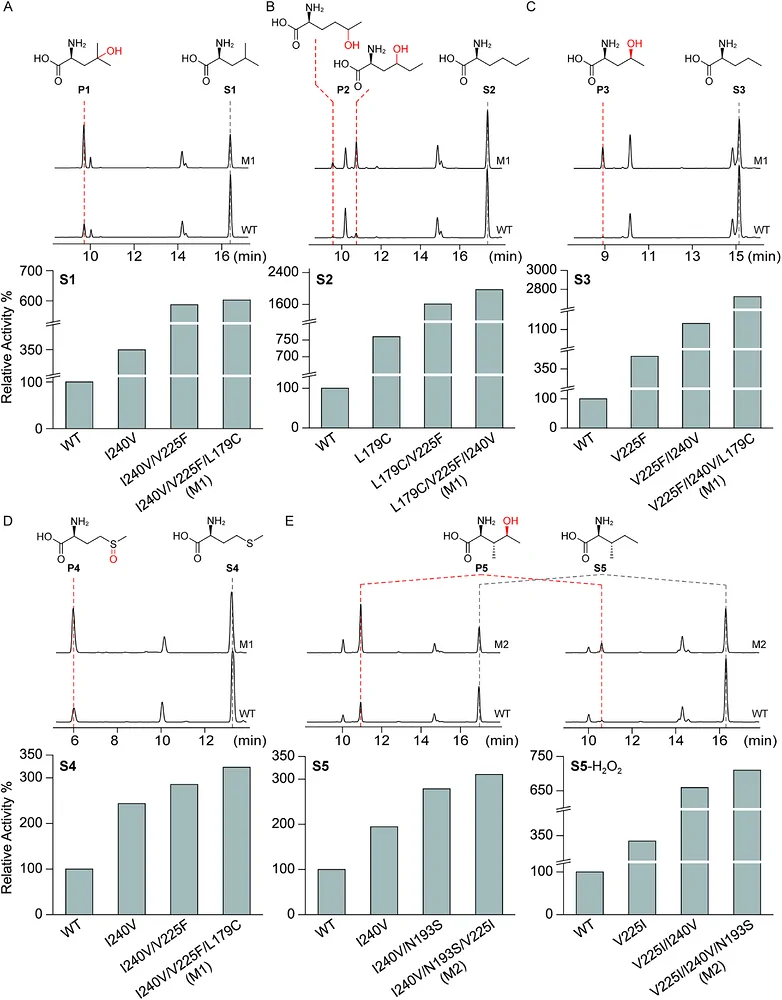
Stepwise evolution of catalytic activity in tunnel hotspot mutants. Catalytic activity of the optimal mutant for **S1** (A), **S2** (B), **S3** (C), **S4** (D), **S5** (E, left), and **S5** with H_2_O_2_ (E, right) in each iteration, including HPLC analysis of each substrate catalyzed by the WT and the optimal single‐point mutant (upper panels), and gradually increasing activity through iterative mutations (lower panels).

**FIGURE 5:**
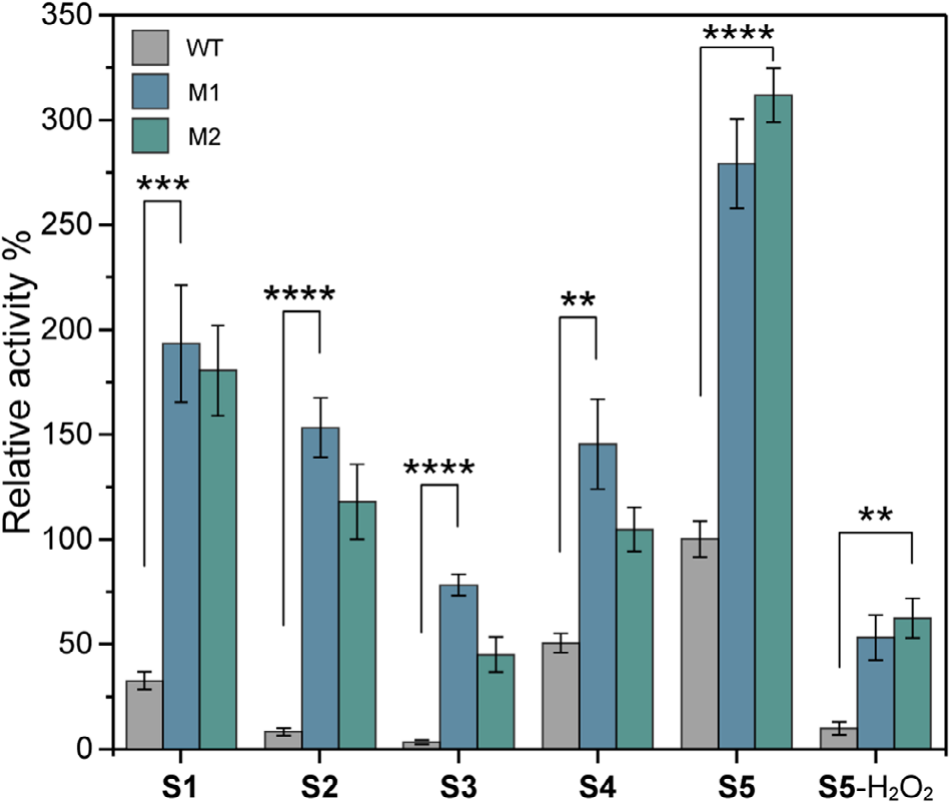
Catalytic performance of WT and mutants M1/M2. Comparison of the catalytic performance of WT IDO and IDO mutants using substrates **S1**–**S5**; **S5** activity was additionally measured under H_2_O_2_ stress. All catalytic activities, including **S5** under H_2_O_2_ stress, were normalized to WT activity toward **S5** (set as 100%). Values represent the averages of three individual determinations. Significant differences between groups (*n* = 3) were determined by one‐way ANOVA followed by Bonferroni multiple comparisons test, with significance levels denoted as *p* < 0.05 (^*^), *p* < 0.01 (^**^), and *p* < 0.001 (^***^), *p* < 0.0001 (^****^).

### 2.4 Kinetics Analysis of O_2_ Transport and H_2_O_2_ Resistance

To investigate whether the tunnel‐engineered mutants regulated amino acid hydroxylation through the O_2_ transport pathway of IDO, we assessed the O_2_ steady‐state kinetics of the WT enzyme and M1 and M2 under varying O_2_ concentrations (Figure 6A,C). As dissolved O_2_ concentrations increased, both M1 and M2 exhibited higher hydroxylation rates and reached catalytic saturation more readily than the WT enzyme. The corresponding kinetic parameters were obtained by Michaelis–Menten fitting (Table 1). M1 and M2 exhibited *k*_cat_ values of 0.036 and 0.037·s^−1^ respectively, representing 1.7‐ and 1.8‐fold increases, respectively, over that of the WT (0.021·s^−1^). Moreover, their *K*_m_ [O_2_] values of 96 µm and 95 µm were significantly lower than that of the WT (346 µm), leading to catalytic efficiencies (*k*_cat_/*K*_m_ [O_2_]) of 374.1 M^−1^·s^−1^ and 390.0 M^−1^·s^−1^, respectively, which represent 6.1‐ and 6.3‐fold increases when compared with that of the WT (61.5 M^−1^·s^−1^). These results indicate that enhanced O_2_ turnover (higher *k*_cat_) and improved apparent O_2_ affinity (lower *K*_m_ [O_2_]) under standard conditions drove the observed increase in hydroxylation activity.

**FIGURE 6:**
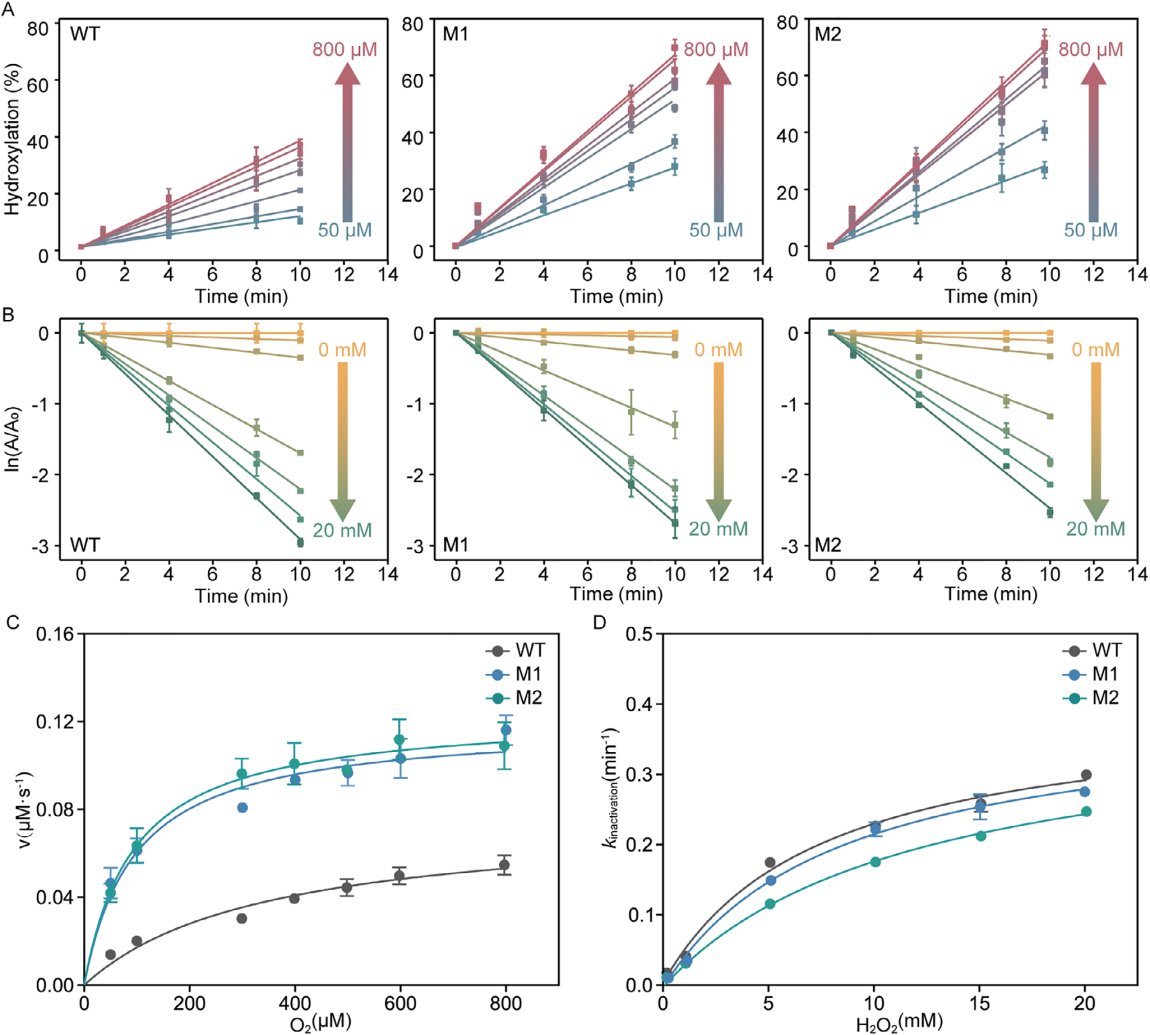
Kinetic and viscosity analyses of O_2_ transport and H_2_O_2_ resistance in WT IDO and engineered variants. (A) Time‐fit lines of hydroxylation activity of WT IDO and engineered variants under different dissolved O_2_ concentrations. (B) Time‐dependent inactivation profiles of WT IDO and engineered variants incubated with different H_2_O_2_ concentrations, and ln(activity treated/untreated) was plotted against time. (C) Steady‐state kinetic parameters under varying O_2_ concentrations. (D) Relative activities of WT IDO and engineered variants in the presence of 15% glycerol. Activities measured in the absence of glycerol were defined as 100%. Data represent mean ± SD (*n* = 3). E) Determination of irreversible inhibition kinetic parameters of WT IDO and engineered variants toward H_2_O_2_.

**TABLE 1:** O_2_ kinetic parameters of WT IDO and optimal mutants.

Enzyme	*k*_cat_ (s^−1^)	*K*_m_ [O_2_] (µm)	*k*_cat_/*K*_m_ [O_2_] (s^−1^·M^−1^)
WT	0.021 ± 0.002	346 ± 47	61.5 ± 0.7
M1	0.036 ± 0.001	96 ± 13	374.1 ± 32.6
M2	0.037 ± 0.004	95 ± 7	390.0 ± 30.2

In addition, solvent viscosity experiments were performed by introducing 15% glycerol into the reaction system to further evaluate the relationship between catalytic performance and O_2_ transport (Figure S12). Since increased solution viscosity reduces O_2_ diffusion efficiency [39], enzyme activity under these conditions is more sensitive to limitations in O_2_ transport. Under viscous conditions, M1 and M2 retained higher relative activities than the WT enzyme, suggesting that the engineered variants possessed more efficient O_2_ accessibility and transport properties. These results further support enhanced O_2_ transport in the engineered variants.

Given that the inhibitory effect of H_2_O_2_ on IDO showed characteristics of an irreversible inhibitor [40], we examined the oxidative stability of the WT and both M1 and M2 after incubating the enzymes with different H_2_O_2_ concentrations over varying time periods, followed by kinetic analysis (Figure 6B,D). The experimental data were fitted to irreversible inhibition kinetics to determine the maximum inactivation rate constant (*k*_max_), the inhibitor concentration causing half‐maximal inactivation (*K*_i_), and the corresponding *k*_max_/*K*_i_ values (Table 2). Lower *k*_max_/*K*_i_ values indicate stronger resistance toward H_2_O_2_‐mediated inactivation. Compared with the WT enzyme, both M1 and M2 exhibited reduced *k*_max_/*K*_i_ values, with M2 showing the most significant improvement (0.032 vs. 0.052 min^−1^·mM^−1^). Notably, the improved oxidative stability was mainly attributable to an increased *K*_i_ rather than reduced *k*_max_, suggesting that the associated mutations potentially reduced H_2_O_2_ accessibility or binding within the active site.

**TABLE 2:** Inhibitory kinetic parameters of H_2_O_2_ in WT, M1, and M2 IDO.

Enzyme	*k*_max_ (min^−1^)	*K*_i_ (mM)	*k*_max_/*K*_i_ (min^−1^·mM^−1^)
WT	0.40 ± 0.02	7.60 ± 0.83	0.052 ± 0.003
M1	0.40 ± 0.03	8.54 ± 0.16	0.046 ± 0.001
M2	0.39 ± 0.03	12.29 ± 1.85	0.032 ± 0.003

These results suggest that tunnel engineering improved O_2_ transport while reducing H_2_O_2_ accessibility to the catalytic center, thereby enhancing both catalytic activity and oxidative resistance.

To further evaluate whether tunnel engineering affected the catalytic utilization of αKG, steady‐state kinetic analysis was performed under varying αKG concentrations (Table S1). The apparent *K*_m_ [αKG] values of M1 and M2 remained comparable to those of the WT enzyme, and no significant decrease in catalytic efficiency was observed after tunnel engineering. These results suggest that the introduced mutations primarily regulated O_2_/H_2_O_2_ transport while maintaining efficient αKG utilization.

### 2.5 Molecular Mechanisms of O_2_ and H_2_O_2_ Transport Regulation by Tunnel Residues

We investigated the molecular mechanisms underlying the enhanced catalytic activity and H_2_O_2_ resistance of the advantageous mutants M1 and M2 through steered molecular dynamics (SMD) simulations of the small‐molecule transport channels most relevant to the mutated sites, T1, T2, and T4 (Figure 1A). Each tunnel was individually probed using O_2_ and H_2_O_2_ as the steered molecules in separate simulations to examine the forces encountered during transport. These simulations revealed pronounced differences between WT IDO and IDO mutants in O_2_ transport through the T1 and T2 tunnels (especially T2), and in H_2_O_2_ transport through the T4 tunnel (Figure 7A), prompting further analysis of the corresponding dissociation processes.

**FIGURE 7:**
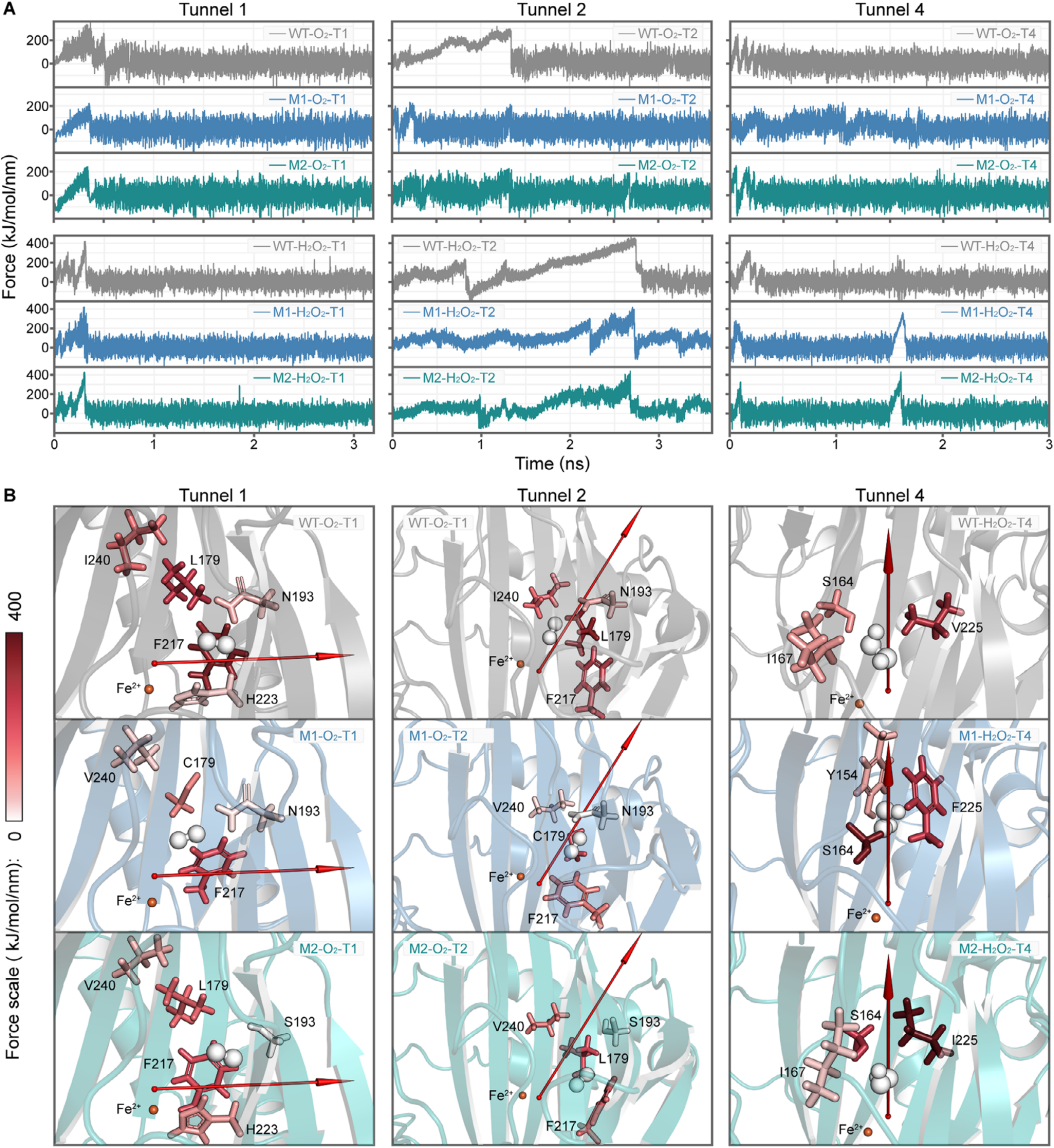
Force profiles and structural analysis. (A) The pulling forces of the small molecules O_2_ and H_2_O_2_ were measured as they were steered out of the active site in WT, M1, and M2 IDO through tunnels T1, T2, and T4, which were directly related to the mutation sites. (B) Representative SMD snapshots taken exactly when the pulling force peaked during the translocation of small molecules from the active site through tunnels T1, T2, and T4. Key residues are shown as sticks, colored according to the pulling force experienced during the simulation.

O_2_ transport through T1 in the WT enzyme was primarily hindered by the bulky side chain of F217, with residues L179 and N193 further reinforcing this spatial barrier and collectively limiting the overall transport efficiency (Figure 7B, WT‐O_2_‐T1). The L179C and N193S substitutions in the two mutants reduced the side‐chain size, provided additional space within the T1 tunnel, and enhanced O_2_ transport (Figure 7B, M1‐O_2_‐T1 and M2‐O_2_‐T1), with no measurable effect on H_2_O_2_ transport (Figure 7A, M1‐H_2_O_2_‐T1 and M2‐H_2_O_2_‐T1).

During outward O_2_ stretching along the T2 tunnel in the WT enzyme, the force of resistance gradually increased to a peak of approximately 300 kJ·mol^−1^·nm^−1^ at around 1.35 ns (Figure 7A, WT‐O_2_‐T2). The small‐molecule transport barrier in the T2 tunnel consisted of residues L179, N193, F217, and I240, with L179 making the largest contribution (Figure 7B, WT‐O_2_‐T2). Substitutions at these positions reduced the side‐chain size and improved O_2_ transport (Figure 7B, M1‐O_2_‐T2 and M2‐O_2_‐T2) with little effect on H_2_O_2_ transport (Figure 7A, M1‐H_2_O_2_‐T2 and M2‐H_2_O_2_‐T2). Although the resistance for H_2_O_2_ transport through T2 was slightly reduced in M1 and M2, H_2_O_2_ transport was still hindered by bulky residues in the outer T2 region (Figure S13). Thus, the engineered variants maintained H_2_O_2_ resistance while facilitating O_2_ transport through T2.

H_2_O_2_ exhibited high permeability through the T4 tunnel in WT IDO, resulting in the highest local resistance from residue V225 during dissociation (Figure 7A, WT‐H_2_O_2_‐T4; Figure 7B, WT‐H_2_O_2_‐T4). In contrast, both M1 and M2 had an enlarged side chain at position 225 that impeded H_2_O_2_ transport (Figure 7B; M1‐H_2_O_2_‐T4 and M2‐H_2_O_2_‐T4). This steric hindrance reduced the probability of H_2_O_2_ accessing the active site, consistent with the increased *K*_i_ values for H_2_O_2_ observed with M1 and M2 (Table 2).

To further validate the proposed tunnel modulation mechanism, the crystal structure of M1 (PDB ID: 26DB) was determined by X‐ray diffraction, and the corresponding data collection and refinement statistics are summarized in Table S2. Due to severe crystal twinning that prevented high‐resolution structure determination, the structure of M2 used for comparison was predicted using Protenix [46] (Figure 8). Structural comparison showed that the O_2_‐T1 and O_2_‐T2 tunnels mainly involved residues 179, 193, 217, and 240. In M1, the L179C mutation increased the *d_179‐217_* and *d_217‐223_* distances, providing additional space for conformational changes of F217 in the O_2_‐T1 tunnel. The I240V mutation further increased the *d_179‐240_* distance, facilitating O_2_ transport through the T2 pathway. Similarly, the N193S mutation in M2 enlarged the upper region of the T1 and T2 tunnels, thereby enhancing O_2_ transport efficiency. In contrast, mutations at residue 225 in both M1 and M2 decreased the *d_146‐226_* distance, resulting in a more constricted H_2_O_2_‐T4 tunnel and thereby limiting H_2_O_2_ transport into the active‐site region. These structural observations were highly consistent with the trends obtained from the SMD analyses (Figure 7).

**FIGURE 8:**
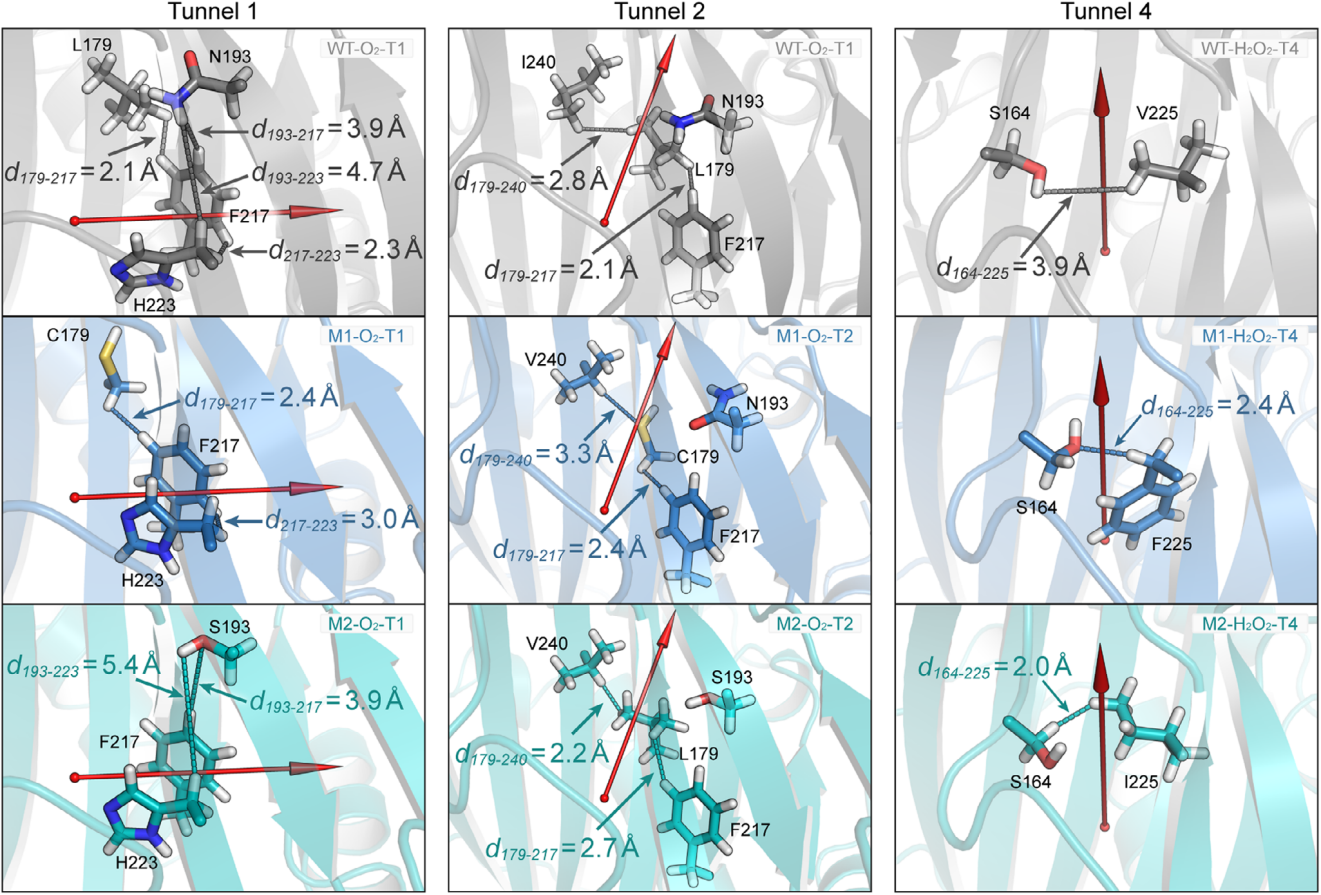
Structural comparison of key tunnel‐lining residues in WT and mutant enzymes. WT (PDB ID: 6LNH) and M1 (PDB ID: 26DB) structures were obtained from X‐ray crystal structures, whereas the M2 structure was predicted using Protenix [46]. The distances shown represent the minimum distances between the indicated residue pairs.

Besides small molecules (O_2_/H_2_O_2_), the IDO reaction system also involves substrate and αKG components. To clarify whether these components contribute to the improved performance of the variants, a detailed discussion was provided. First, a recent study indicated that substrate anchoring and transport in IDO occur predominantly along the flexible‐loop side of the active pocket, distal to the DSBH structural motif [47], whereas the tunnel mutations introduced in this work reside within the DSBH structural motif, indicating that the observed activity enhancement of the variants is unlikely to be substrate‐related. Additionally, kinetic measurements for αKG revealed comparable *K*_m_ and *k*_cat_ values among WT, M1, and M2, suggesting that these mutations do not substantially affect αKG utilization. In contrast, kinetic analyses of O_2_ and H_2_O_2_ revealed that, relative to WT, the M1 and M2 variants exhibit markedly optimized *K*_m_ and *k*_cat_ values for O_2_, alongside enhanced resistance to H_2_O_2_‐mediated inactivation. Further mechanistic investigations underscored that these variants primarily modulate the accessibility of small molecules (O_2_ and H_2_O_2_) by altering the side‐chain sizes of key tunnel residues, thereby facilitating O_2_ transport while restricting H_2_O_2_ entry.

### 2.6 In Vitro Biosynthesis and Cascade Reaction of 4‐Hydroxyisoleucine (4‐HIL)

To further evaluate the performance of the most favorable mutants in hydroxylated product biosynthesis and H_2_O_2_‐resistance capability in a one‐pot multi‐enzyme cascade system, we employed the mutant M2 (which exhibited the highest activity toward Ile and the strongest H_2_O_2_ resistance) to produce 4‐HIL from high‐concentration Ile (500 mm) in a 5 mL reaction mixture. WT IDO served as the control.

For Ile hydroxylation, the reaction mixtures contained 500 mm substrate Ile and 1 mg·L^−1^ purified IDO or its variants as hydroxylation catalysts (Figure 9A). M2 completely converted 500 mm Ile within 10 h, whereas WT IDO required over 25 h for full conversion. The space–time yield (STY) increased from 2.89 g·L^−1^·h^−1^ to 7.29 g·L^−1^·h^−1^, representing approximately a 2.5‐fold improvement, demonstrating the powerful biosynthetic capability of the tunnel‐engineered mutant for efficient substrate hydroxylation.

**FIGURE 9:**
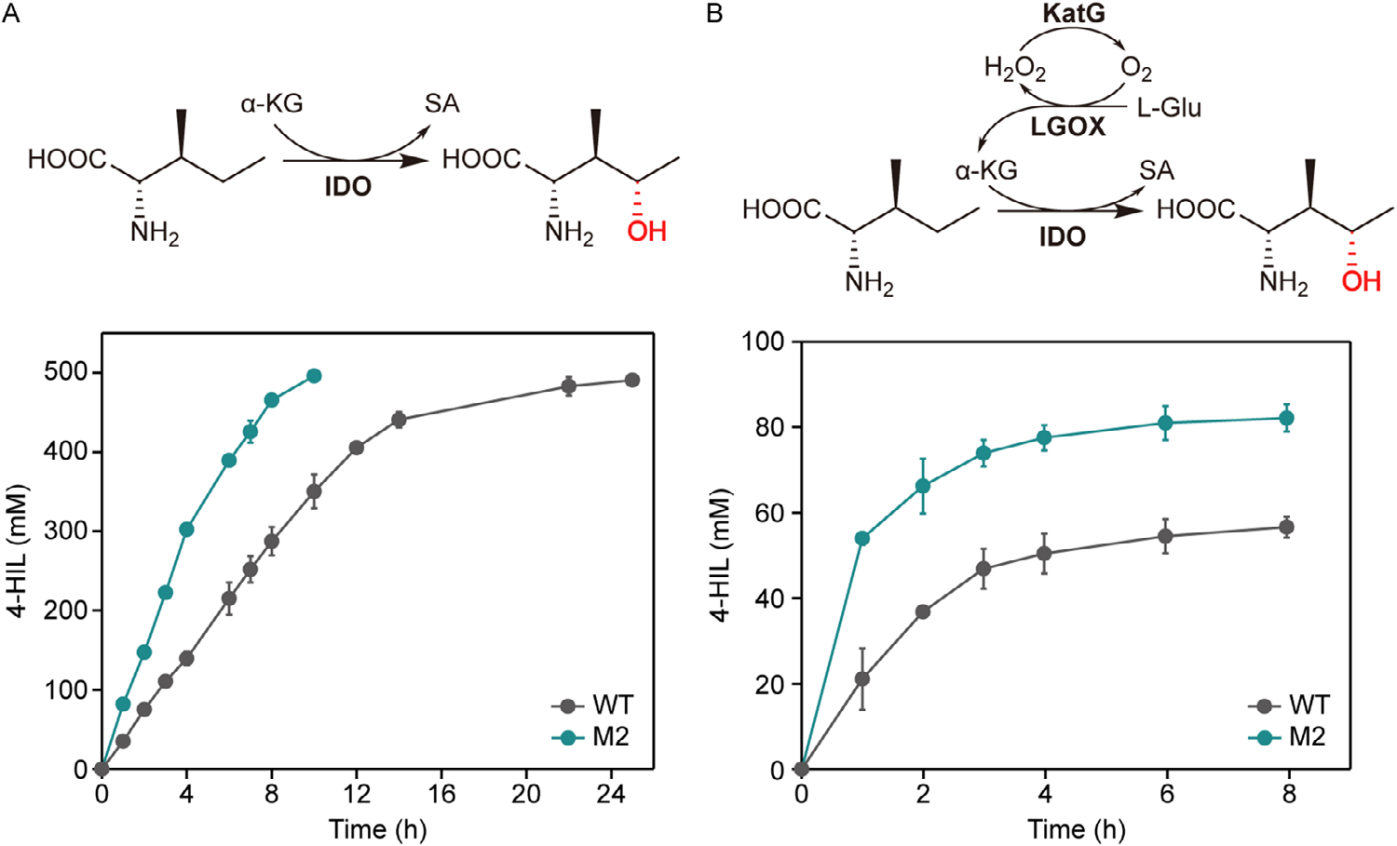
In vitro and one‐pot cascade reaction biosynthesis of 4‐HIL. (A) In vitro biosynthesis of 4‐HIL by IDO and its variants. (B) One‐pot cascade biosynthesis of 4‐HIL by IDO and variants with the co‐substrate αKG supplied. Values represent the averages of three individual determinations.

During one‐pot, single‐step cascade biosynthesis, the reaction mixtures contained both αKG supply and the hydroxylation components, including purified LGOX, KatG, and IDO (or its variants) as catalysts (Figure 9B). During the first 3 h of the reaction, 4‐HIL gradually accumulated, with M2 exhibiting a higher reaction rate than the WT. After 4 h, only a small amount of the product was generated with either the mutant or WT reaction systems. At this point, the M2 reached an STY of 2.81 g·L^−1^·h^−1^, representing a 1.5‐fold increase compared with that of the WT (1.83 g·L^−1^·h^−1^). Although the tunnel‐engineered mutant showed significantly enhanced catalytic performance in the cascade reaction, the overall conversion yield remained at only 15.3% at a substrate concentration of 500 mm. This incomplete conversion was attributable to two main factors: (1) Fe(II) can inhibit the αKG‐supplying enzyme LGOX (Figure S14). Consistently, the generation rate of αKG was relatively low in our cascade system, leading to a gradual increase in its concentration. This inhibitory effect likely hindered the supply of αKG, thereby limiting the cascade‐reaction efficiency. (2) The tunnel‐engineered mutant M2 exhibited a greater antioxidant capacity than the WT at a defined H_2_O_2_ concentration of 10 mm. Nevertheless, during the cascade reaction, H_2_O_2_ and αKG were generated simultaneously at a 1:1 molar ratio. Monitoring of H_2_O_2_ concentration further revealed its dynamic accumulation throughout the reaction process (Figure S15). As the reaction progressed, the continuous accumulation of H_2_O_2_ likely exceeded the tolerance threshold of the mutant, resulting in failure of its antioxidant defense and a rapid loss of enzyme activity. The model offers a plausible rationale for the cessation of 4‐HIL accumulation despite the presence of residual αKG in the system (Figure S16).

The industrial application of IDO has long posed two major challenges: enhancing its catalytic activity and increasing the supply of αKG. To address the first challenge, a previous study developed an engineered *Bacillus subtilis* IDO variant, I162T/T182N, which achieved complete conversion of 228 mM Ile, with a STY of 3.67 g·L^−1^·h^−1^ (80.8 g·L^−1^·d^−1^) and a conversion rate of 91% at 1 m Ile [48]. To overcome the second challenge, another report established a one‐pot, two‐step cascade system combining LGOX/CAT with IDO: αKG was accumulated via LGOX/CAT (with H_2_O_2_ as a byproduct) in the first step to support efficient hydroxylation by IDO in the second step, ultimately yielding 465 mM 4‐HIL at 93% conversion [21]. In this work, small‐molecule tunnel engineering was employed to simultaneously address both challenges, resulting in a substantial enhancement of IDO catalytic activity (2.5‐fold increase in STY) and improved H_2_O_2_ resistance. This engineered result supports an attempt to establish a one‐pot, single‐step LGOX/CAT/IDO cascade system, although the resistance capacity of the variants, assessed under 10 mm H_2_O_2_, was insufficient to fully withstand oxidative stress caused by dynamically accumulated H_2_O_2_ (theoretically up to 500 mm), thereby preventing complete substrate conversion in this one‐pot, single‐step cascade. Nevertheless, the improved performance of M1 and M2 indicates that small‐molecule tunnel engineering may present a promising strategy for the large‐scale production of IDO hydroxylation reactions and the optimization of cascade systems.

## 3 Conclusion

We devised a tunnel‐guided strategy combining RAMD simulations and protein structuromics to design an optimized IDO mutant. This mutant demonstrated substantially higher activity toward aliphatic amino acid substrates and enhanced resistance to H_2_O_2_, resulting in improved enzyme performance and production efficiency. The obtained advantageous variants showed enhanced activity for all five substrates tested, with a maximum increase of 27.2‐fold, and acquired antioxidant capacity, exhibiting up to 7.2‐fold higher activity than the WT in the presence of H_2_O_2_. Specifically, the most effective variant achieved a 6.3‐fold increase in catalytic efficiency (*k*_cat_/*K*_m_ [O_2_]) and a 1.6‐fold improvement in oxidative stability (*k*_max_/*K*_i_) relative to those of the WT. These improvements translated into robust performance in in vitro biosynthesis for 4‐HIL production, where the variants achieved complete conversion of 500 mm Ile with a maximum STY of 7.29 g·L^−1^·h^−1^, representing a 2.5‐fold improvement over the WT. In multi‐enzyme cascade reactions, although excessive H_2_O_2_ accumulation at high substrate concentrations limited complete conversion, the variants still reached a STY of 2.81 g·L^−1^·h^−1^, representing 1.5 times that of the WT. Notably, the beneficial sites converged on several key tunnel residues, including L179, N193, V225, and I240, and steady‐state kinetics and SMD simulations confirmed that mutations at these sites enhanced O_2_ entry into the active pocket while reducing H_2_O_2_ accessibility through steric hindrance.

Previous protein‐engineering efforts with αKGDs have mainly concentrated on residues within the substrate‐binding pocket, in order to improve enzyme activity, selectivity, or substrate specificity. For example, reshaping the binding pocket enhanced lysine hydroxylase activity, substituting substrate‐positioning residues modulated regioselectivity in IDO [13, 41], and mutating hydrophobic residues lining the active pocket reprogrammed the substrate specificity and reactivity of SptF [42, 43]. In contrast, we shifted the focus to underexplored tunnels that govern O_2_ and H_2_O_2_ access. Additionally, we employed enhanced sampling via RAMD simulations to explore the small‐molecule tunnels and identify key tunnel residues. Protein structuromics was combined for conservation analysis of the identified residues to rapidly narrow the mutation library. This combined method provides an efficient computational strategy for identifying critical residues and rational design in enzyme engineering.

In summary, the optimization of key tunnel residues successfully enhanced the enzymatic activity and H_2_O_2_ resistance, highlighting the efficacy of small‐molecule tunnel engineering in modulating enzymatic performance. The computational framework for small‐molecule tunnels employed here can be further extended to engineering other O_2_‐dependent industrial enzymes [44], providing insights into small‐molecule transport mechanisms within enzyme architectures and opening new avenues for rationally developing redox biocatalysts.

## 4 Experimental Section

### 4.1 Structural Modeling and RAMD Simulations of IDO‐Ligand Complexes

We modeled IDO–ligand complexes containing a substrate (Ile as a representative), co‐substrate, and separately coordinated O_2_ and H_2_O_2_ using Protenix [46] (a full reproduction of AlphaFold3).

The protonation states of the proteins were calculated using the H++ web server [49]. The AMBER ff14SB force field was employed for all simulations [50], while the parameters for the coordinated Fe(II) center were generated based on DFT/B3LYP/6‐31G(d,p) and HF/6‐31G* quantum chemical calculations. Ligand topology parameters were generated using ACPYPE [51] based on the GAFF2 force field. Ligand atomic charges were described using the advanced restrained electrostatic potential charge model (RESP2) [52] and calculated using the Multiwfn program [53, 54]. The complexes were placed in a cubic simulation box with periodic boundary conditions, ensuring a minimum distance of 1.2 nm between the protein atoms and the box boundaries. The systems were solvated using the TIP3P water model, and counterions (Na^+^ and Cl^−^) were added to neutralize the total charge. Energy minimization was performed using the steepest descent algorithm (50 000 steps) to eliminate unreasonable contacts. Subsequently, NVT equilibration was carried out at 300 K. During equilibration, hydrogen‐containing bonds were constrained using the LINCS algorithm, long‐range electrostatic interactions were treated using the particle mesh ewald (PME) method, and temperature coupling was controlled using the V‐rescale thermostat. This was followed by NPT equilibration at 300 K, in which pressure coupling was controlled using the Berendsen method, while the parameters for bond constraints, electrostatic treatment, and temperature coupling were kept identical to those used in the NVT equilibration step.

Subsequently, we performed RAMD simulations to explore O_2_ and H_2_O_2_ transport pathways within the protein complexes. A constant external force (14 kcal·mol^−1^·Å^−1^) with random directions was applied to the ligands, O_2_ and H_2_O_2_. For every 100 fs of simulation time, the displacement of the O_2_/H_2_O_2_ center of mass was measured. When the displacement smaller than 0.025 Å, the original force direction was maintained; otherwise, a new random direction was reapplied [55, 56, 57]. The simulations were terminated when the displacement of the center‐of‐mass of O_2_/H_2_O_2_ from its initial position exceeded 40 Å. To ensure sufficient conformational sampling, each system underwent 64 RAMD simulations comprising 16 independent replicas initiated from equilibrated snapshots with randomly assigned initial velocities and repeated four times. All simulations were performed using GROMACS 2024.2 software [58].

### 4.2 Site‐Directed Mutagenesis

Site‐directed point mutations were introduced into IDO in the pET‐28a vector using primers containing the required codons and PrimeSTAR DNA polymerase (Takara Bio, Shiga, Japan) for whole‐plasmid polymerase chain reaction (PCR) amplification. The following PCR program was used: 98°C for 1 min; 30 cycles of 98°C for 30 s, 55°C for 15 s, and 72°C for 2 min; followed by 72°C for 10 min and 16°C for 5 min. The PCR products were analyzed via agarose gel electrophoresis, and the plasmid templates were digested with DpnI (Takara Bio). The digestion products were purified using a PCR purification kit (Tiangen Biotech Co., Ltd.), and the DNA concentration was measured using a NanoDrop 8000 spectrophotometer (Thermo Scientific). The purified plasmids were subsequently transformed into E. coli BL21(DE3) cells, and the mutants were confirmed via DNA sequencing (Sangon Biotech, Shanghai, China).

### 4.3 Protein Expression

The pET‐28a plasmid encoding 6×His‐tagged IDO (WT and mutants) was transformed into *E. coli* BL21(DE3) for recombinant protein expression. Cells were cultured in LB medium with kanamycin (50 µg·mL^−1^) at 37°C, 200 rpm until the OD value at 600 nm reached 0.6–0.8. Isopropyl β‐d‐1‐thiogalactopyranoside was added to a final concentration of 0.1 mM, and the mixture was incubated at 17°C with shaking at 200 rpm for 12–14 h to induce protein expression. The cells were harvested using a microplate centrifuge and stored at −20°C until further purification.

### 4.4 Protein Purification

The cells were resuspended in a buffer (50 mm Tris‐HCl, 150 mm NaCl, pH 7.5) and lysed with TieChui *E. coli* lysis buffer (ACE Biotech, Changzhou, China) at a 1:10 ratio (lysis buffer to resuspension volume). The supernatant was collected via centrifugation (15,000 × *g* for 30 min) and filtration (0.45 µm). The His‐tagged target protein was purified using gravity‐flow Ni‐NTA columns (Sangon Biotech Co. Ltd., Shanghai, China). The purification procedure included column equilibration and sample application using a loading buffer (20 mm Tris‐HCl, 100 mm NaCl, pH 7.5) and protein collection with an elution buffer (20 mm Tris‐HCl, 100 mm NaCl, 300 mm imidazole, pH 7.5).

### 4.5 Hydroxylation‐Activity Assay

Each reaction mixture (300 µL) contained 10 mm amino acid substrate, 1.5 mm FeSO_4_·7H_2_O, 10 mm l‐ascorbic acid, and 10 mm αKG in 20 mm Tris‐HCl buffer (pH 7.0). For the oxidative stress‐tolerance assays, H_2_O_2_ was added to the Ile reaction system at a final concentration of 10 mm. Reactions were initiated by the addition of purified enzyme (0.1 mg·mL^−1^) and incubated at 30°C and 1000 rpm in a Thermomixer Comfort incubator (Eppendorf, Hamburg, Germany). The reactions were terminated by boiling for 10 min, and the hydroxylated products were analyzed via HPLC. The amount of enzyme required to generate 1 µm hydroxy amino acid/min at 30°C was defined as one unit (U) of enzymatic activity.

### 4.6 O_2_ Steady‐State Kinetics

O_2_ steady‐state kinetic assays for IDO were performed using Atmosbags (Sigma‐Aldrich). The defined O_2_ concentrations were established by adjusting the flow rates of O_2_ and N_2_ in the buffer (20 mm Tris‐HCl, pH 7.0), and the resulting O_2_ concentrations were monitored using a trace oxygen meter (PreSens, Germany). All other reaction conditions were maintained as in the standard enzyme activity assay, with reactions terminated at 1, 5, 8, or 10 min. The data were fitted using the Michaelis–Menten equation.

### 4.7 H_2_O_2_‐Induced Irreversible Inactivation Kinetics

Purified enzyme samples were incubated with different concentrations of H_2_O_2_ at 30°C. Residual enzyme activity was measured at predetermined time intervals (1 min, 4 min, 8 min, and 10 min) under standard enzyme activity assay conditions. The natural logarithm of the residual activity relative to that of the untreated control was plotted against the incubation time, with the slope of the linear fit representing the observed first‐order inactivation rate constant. The inactivation rate was then plotted against the H_2_O_2_ concentration and fitted to the Michaelis–Menten equation to determine the maximum inactivation rate constant (*k*_inactivation_) and the *K*_i_. The *k*_inactivation_/*K*_i_ ratio was used as an indicator of the relative resistance of IDO and its mutants to H_2_O_2_‐induced oxidative inactivation.

### 4.8 HPLC Analysis of Hydroxy Amino Acids

Each 250 µL diluted hydroxylated amino acid reaction mixture was mixed with 250 µL H_3_BO_3_ buffer (200 mm, pH 9.2) and 500 µL Fmoc‐Cl (10 mm) for derivatization. Each mixture was incubated at 25°C for 10 min, and the reaction was terminated with 500 µL of adamantane (40 mM). Quantitative analysis was conducted using a Waters 2695 HPLC system and a Diomansil C18 column (4.6 × 250 mm). Mobile phase A comprised NaAc‐HAc (50 mm, pH 4.2) and acetonitrile at a 90:10 (v/v) ratio; mobile phase B comprised NaAc‐HAc (50 mm, pH 4.2) and acetonitrile at a 20:80 (v/v) ratio. Gradient elution was performed at a flow rate of 1 mL·min^−1^, and the Fmoc‐Cl derivatives of the amino acids were detected at 263 nm.

### 4.9 SMD Simulations

SMD simulations were prepared following the same protocol used for standard MD simulations. During the simulations, the small molecules (O_2_ or H_2_O_2_) were subjected to constant velocity pulling along the vector, defined by the starting and ending point coordinates of the tunnel identified through the RAMD. The pulling type was set to the umbrella method, the force constant for pulling was 1000 kJ·mol^−1^·nm^−1^, and the pulling velocity was 0.005 nm·ps^−1^. The SMD simulations were conducted under the NVT ensemble to prevent box size fluctuations from affecting the pulling behavior. The simulations employed a time step of 2 fs and were terminated when the pulled molecule reached the designated position. The coordinates of the molecule and the corresponding average pulling force were recorded every 100 steps.

### 4.10 Crystallization and Structure Determination of IDO M1

To determine the crystal structure of IDO M1, IDO M1 was expressed in *E. coli* BL21(DE3) cells as an N‐terminal His‐SUMO fusion protein. Wet cell paste was resuspended in lysis buffer (25 mm Tris‐HCl and 150 mm NaCl (pH 7.5), treated with lysozyme (5 mg·g^−1^ final concentration) on ice for 30 min, and disrupted by sonication (43 cycles of 3 s bursts) using a VCX130 ultrasonic processor (SONICS & MATERIALS, Inc., Newtown, USA). Following centrifugation (18,000 rpm, 60 min, 4°C), the supernatant was incubated with Ni‐NTA resin (Cytiva, Massachusetts, USA) for 30 min on ice. After the cell lysate was drained by gravity, the column was washed with 10 column volumes of washing buffer (25 mm Tris‐HCl, 150 mm NaCl, 10 mm imidazole, pH 7.5) to remove non‐specific contaminants. The target fusion protein was eluted using an elution buffer supplemented with 100 mm imidazole.

Crystallization was performed using the hanging‐drop vapor diffusion method at 20°C. Building upon the previously reported conditions for IDO WT (PDB ID: 6LNH), the purified protein was concentrated to approximately 8 mg·mL^−1^. Rod‐like crystals were observed after 1 day from a reservoir solution consisting of 19% (w/v) PEG 3,350, 5% (v/v) Tacsimate (pH 7.0), 0.1 m HEPES (pH 7.5). The crystals were cryoprotected by adding glycerol into crystallization solution to reach a final concentration of 20% (v/v) before being flash‐cooled in liquid nitrogen. Diffraction datasets were collected on the BL02U1 beamline at the Shanghai Synchrotron Radiation Facility (SSRF) at 100 K and processed using XDS [59]. Crystallographic statistics are summarized in Table S2.

The crystal structure of apo IDO M1 was determined by molecular replacement using Phaser as implemented in the Phenix suite of programs [60], using the structure of 6LNH as the search model. The initial solution was further improved using iterative cycles of manual model building in COOT [61], followed by refinement using phenix.refine [62, 63]. All structural figures were rendered using PyMOL.

### 4.11 Biosynthesis and Cascade Reaction of 4‐HIL

The biosynthesis of 4‐HIL, the hydroxylated product of Ile, was conducted in a 5 mL reaction system containing 500 mm αKG, 500 mm Ile, 5 mm FeSO_4_, 50 mm Vc, and 1 g·L^−1^ IDO (in 50 mm Tris‐HCl buffer, pH 7.0). The reaction proceeded at 30°C with shaking at 200 rpm.

The enzymatic cascade for 4‐HIL biosynthesis was performed in a 5 mL reaction system containing 500 mm l‐Glu, 0.5 mg·mL^−1^ LGOX, and 2 mg·L^−1^ catalase (200 U·mg^−1^) to provide αKG for IDO. The reaction mixture also included 500 mm Ile, 5 mm FeSO_4_, 50 mm ascorbic acid, and 1 g·L^−1^ IDO in 50 mm Tris‐HCl buffer (pH 7.0). The reactions were performed at 30°C with shaking at 200 rpm.

### 4.12 Statistical Analysis

Statistical comparisons between groups were performed using one‐way analysis of variance (ANOVA) followed by Bonferroni's multiple comparisons test. Differences between groups were considered statistically significant at the following levels: *p* < 0.05 (^*^), *p* < 0.01 (^**^), and *p* < 0.001 (^***^), *p* < 0.0001 (^****^). All statistical analyses were performed using GraphPad Prism, and results are presented as means of three independent experiments (*n* = 3).

## Author Contributions

H.(Huan) L. and L. W. contributed equally to this work. Huan L., Y. N., and Y. X. conceived and designed the experiments. H.(Huan) L., H.(Hu) L., and J. G. constructed mutants. H.(Huan) L., L. W., H.(Hu) L., D. Z., and S. X. purified the enzymes and performed activity characterization. L. Mao and X. Wang performed protein crystallization, X‐ray data collection, and crystal structure determination. L.W., Huan L., and S. Z. performed random acceleration molecular dynamics simulations and conservation analysis. H.(Huan) L., L. W., and Y. N. wrote the manuscript. All authors have given approval to the final version of the manuscript.

## Conflicts of Interest

The authors declare no conflict of interest.

## Supporting information




**Supporting File**: advs76328‐sup‐0001‐SuppMat.docx.

## Data Availability

The data that support the findings of this study are available from the corresponding author upon reasonable request.
