# Supplementary material for: Computation‐Guided Tunnel Engineering Enhances O2 Transport and H2O2 Resistance of Fe(II)/α‐Ketoglutarate‐Dependent Dioxygenases
Source: Adv Sci (Weinh). 2026 Jul 6;13(53):e76328. doi: 10.1002/advs.76328 (PMC13335103; doi:10.1002/advs.76328)
Supplement: Supplementary file 1 [file ADVS-13-e76328-s001.docx]

Supporting Information

Computation-Guided Tunnel Engineering Enhances O_2_ Transport and H_2_O_2_ Resistance of Fe(II)/α-Ketoglutarate-Dependent Dioxygenases

Huan Liu, Lunjie Wu, Liying Mao, Songyin Zhao, Hu Liu, Shiyi Xin, Donglin Zhao, Jie Gu, Yan Xu, Xinye Wang*, Yao Nie*

H.(Huan) Liu, L. Wu, S. Zhao, H.(Hu) Liu, S. Xin, D. Zhao, J. Gu, Y. Xu, Y. Nie

Lab of Brewing Microbiology and Applied Enzymology, School of Biotechnology, The Key Laboratory of Industrial Biotechnology, Ministry of Education, Jiangnan University, Wuxi 214122, China; Key Laboratory of Industrial Synthetic Biology of Jiangsu Province, Jiangnan University, Wuxi 214122, Jiangsu, China.

E-mail: ynie@jiangnan.edu.cn (Y. Nie); Tel.: +86-510-85197760; Fax: +86-510-85918201

Y. Xu
State Key Laboratory of Food Science and Technology, Jiangnan University, 1800 Lihu Road, Wuxi 214122, China.

L. Wu

Institute of Biopharmaceutical and Health Engineering, Tsinghua Shenzhen International Graduate School, Tsinghua University, Shenzhen 518055, China.

L. Mao, X. Wang

School of Life Sciences, Ludong University, Yantai, Shandong 264025, China.

E-mail: xinyewang@ldu.edu.cn (X. Wang)

**Supporting Figures**

**Figure S1.** The structure of IDO-ligand (O_2_ and H_2_O_2_) complexes modeled by artificial intelligence tools (A), and analysis of O_2_ and H_2_O_2_ transport tunnels in IDO by RAMD simulations (B).

**Figure S2.** Multiple sequence alignment of representative protein sequences from each cluster (A), phylogenetic analysis based on protein sequences (B).

**Figure S3.** Protein structure alignment of representative proteins in each cluster based on FoldMason (A)，sequence alignment corresponding to protein structure alignment results (B), phylogenetic analysis based on protein structure (C), structural alignment view (D).

**Figure S4.**  Iterative variants in descending order of activety for the substrate **S1**.

**Figure S5.**  Iterative variants in descending order of activety for the substrate **S2**.

**Figure S6.**  Iterative variants in descending order of activety for the substrate **S3**.

**Figure S7.**  Iterative variants in descending order of activety for the substrate **S4**.

**Figure S8.** Iterative variants in descending order of activety for the substrate **S5**.

**Figure S9.** Iterative variants in descending order of activety for the substrate **S5** with H_2_O_2_.

**Figure S10.** The SDS-PAGE analysis of the top 5 iterative variants toward **S1**-**S5** and **S5**-H_2_O_2_.

Figure S11. Residual activities of WT, M1, and M2 toward 10 mM S5 under different H_2_O_2_ concentrations (0-20 mM). All catalytic activities were normalized to WT activity toward S5 (set as 100%). Data represent mean ± SD (n = 3).

**Figure S12.** Effect of solvent viscosity on enzyme activity toward **S5**. Enzyme activity was measured in the absence and presence of 15% glycerol. The activity without glycerol was defined as 100%, and residual activity in 15% glycerol was calculated as relative activity. Data represent mean ± SD (n = 3).

Figure S13. Steric hindrance of outer T2-tunnel residues toward H_2_O_2_ transport in the WT enzyme.

**Figure S14**. Inhibition of LGOX activity by Fe(II). Data represent mean ± SD (n = 3).

**Figure S15**. Accumulation of H_2_O_2_ in the IDO-LGOX cascade reaction. Data represent mean ± SD (n = 3).

**Figure S16.** The concentration of α-KG gradually increases in the cascade system.

**Table S1**. αKG kinetic parameters of WT IDO and optimal mutants.

| Enzyme | *k*_cat_ (s^-1^) | *K*_m_ [αKG] (mM) | *k*_cat_/*K*_m_ [αKG] (s^-1^·mM^-1^) |
| --- | --- | --- | --- |
| WT | 0.466 ± 0.018 | 0.481± 0.096 | 0.97 ± 0.20 |
| M1 | 0.564 ± 0.019 | 0.666 ± 0.110 | 0.85 ± 0.14 |
| M2 | 0.537 ± 0.018 | 0.513 ± 0.088 | 1.05 ± 0.18 |

**Table S2.** Crystallographic data collection and refinement statistics of the IDO M1 mutant.

|  | IDO M1 |
| --- | --- |
| **Data Collection** |  |
| Wavelength (Å) | 0.9791 |
| Resolution range (Å) | 37.72 - 2.0  (2.071 - 2.0) |
| Space group | C 1 2 1 |
| Unit cell parameters  a, b, c (Å)  α, β, γ (°) | 141.4, 54.1, 133.5,  90, 90.1, 90 |
| Unique reflections | 67527 (6586) |
| Multiplicity | 3.3 (2.6) |
| Completeness (%) | 98.11 (96.61) |
| Mean I/σ(I) | 16.40 (1.85) |
| R_merge_ | 0.042 (0.527) |
| R_meas_ | 0.050 (0.662) |
| R_pim_ | 0.027 (0.395) |
| CC_1/2_ | 0.999 (0.71) |
| **Refinement** |  |
| R_work_ | 0.18 (0.26) |
| R_free_ | 0.23 (0.31) |
| Number of non-hydrogen atoms | 7474 |
| Macromolecules | 7087 |
| Ligands | 0 |
| Water | 387 |
| Protein residues | 865 |
| RMS bonds (Å) | 0.007 |
| RMS angles (°) | 0.88 |
| **Ramachandran plot** |  |
| Favoured regions (%) | 98.21 |
| Allowed regions (%) | 1.67 |
| Outliers (%) | 0.12 |
| Average B-factors (Å^2^) | 47.50 |
| Macromolecules | 47.46 |
| Water | 48.26 |
